# Association between cardiac arrhythmia before pregnancy and gestational diabetes: a nationwide population-based study in Korea

**DOI:** 10.4178/epih.e2023103

**Published:** 2023-12-04

**Authors:** You-Jung Choi, Won Young Wi, Geum Joon Cho, Jin Oh Na

**Affiliations:** 1 Division of Cardiology, Department of Internal Medicine, Korea University Guro Hospital, Seoul, Korea; 2 Department of Obstetrics and Gynecology, Korea University Guro Hospital, Seoul, Korea

**Keywords:** Gestational diabetes, Arrhythmia, Cardiovascular disease

## Abstract

Given the higher prevalence of cardiac arrhythmias in individuals with diabetes, we investigated the relationship between cardiac arrhythmias and the incidence of gestational diabetes (GDM). This retrospective cohort study utilized data from the Korean Health Insurance Service database, encompassing 1,113,729 women who gave birth between January 2007 and December 2015. After excluding those who did not undergo National Health Screening tests within 1 year prior to pregnancy, those with multifetal pregnancies, and those diagnosed with diabetes, we analyzed 365,880 singleton pregnancies without a history of diabetes. Of these, 3,253 (0.9%) had cardiac arrhythmias, including premature extra beats, supraventricular tachyarrhythmias, and/or atrial flutter/fibrillation. GDM occurred in 31,938 (8.7%) subjects during pregnancy, and was more prevalent in women with cardiac arrhythmia than in those without (14.9 vs. 8.7%, p<0.001). In the multivariate analysis, the association between cardiac arrhythmia and GDM remained statistically significant (adjusted odds ratio, 1.78; 95% confidence interval, 1.61 to 1.97; p<0.001). Subgroup analysis revealed that the risk of GDM was consistently statistically significant in subjects with cardiac arrhythmia, regardless of age, body mass index, and the presence or absence of chronic hypertension. Therefore, cardiac arrhythmias before and during pregnancy appear to be associated with an increased risk of developing GDM.

## GRAPHICAL ABSTRACT

[Fig f2-epih-45-e2023103]

## INTRODUCTION

Gestational diabetes (GDM) impacts roughly 7% of all pregnancies, equating to a global prevalence of over 200,000 cases each year [1]. GDM increases the risk of complications for the mother, particularly hypertensive disorders of pregnancy like preeclampsia, as well as complications for the fetus, notably macrosomia and birth injury [2]. In recent years, there has been a growing awareness of risk factors linked to GDM [3]. These include ethnicity, maternal age over 35 years, obesity, lack of physical activity, and a family history of diabetes [4].

Premature extra beats and supraventricular tachyarrhythmias are the most common cardiac complications during pregnancy, affecting up to 50% of pregnant women [5,6]. Cardiac arrhythmias are more frequently observed in individuals with diabetes mellitus compared to those without the condition [7]. However, there is a lack of substantial data regarding the relationship between cardiac arrhythmia and GDM. Consequently, we conducted a study to explore the potential connection between cardiac arrhythmia prior to and during pregnancy and the onset of GDM.

## MATERIALS AND METHODS

### Study design and data collection

This retrospective cohort study utilized the Korean National Health Insurance Service (NHIS)-Health Screening Cohort databases, which cover 97% of the Korean population. This integrated, time-series database comprises both medical claim record data and electronic resident registration data. The database is publicly accessible via the Healthcare Bigdata Hub (https://nhiss.nhis.or.kr).

### Study population

The domestic procedure codes for delivery (R3131, R3133, R3136, R3138, R3141, R3143, R3146, R3148, R4361, R4362, R4380, R4507, R4508, R4509, R4510, R4514, R4516-R4520, R5001, and R5002) were employed to identify all women who had given birth during the study period. We analyzed 1,113,729 singleton pregnancies between 2007 and 2015. We excluded subjects who had not undergone the national health screening test within 1 year prior to pregnancy (n= 723,575), those with multifetal pregnancies (n= 17,683), and individuals diagnosed with diabetes (as per the International Classification of Diseases, 10th revision [ICD-10] codes E08, E09, E10, E11, and E13) and/or those with a fasting blood glucose level of 126 mg/dL or higher at the health screening test conducted within 1 year before pregnancy (n= 6,591).

### Cardiac arrhythmia

Arrhythmias were classified into 5 categories: lethal arrhythmias, premature beats, paroxysmal tachycardia, atrial flutter/fibrillation, and atrioventricular block. Specific codes were assigned to each category, determined in consultation with at least 2 cardiologists (Table 1). After excluding lethal arrhythmia and atrioventricular block, the remaining categories—premature beats (atrial and ventricular premature beats) and supraventricular tachyarrhythmias (paroxysmal tachycardia and atrial fibrillation/flutter)—were collectively defined as cardiac arrhythmia for the purposes of this study. These definitions of arrhythmia were validated in a previous study [8].

### Primary outcome

The primary outcome was incident GDM, which was defined as having at least 1 claim with ICD-10 codes of O244 or O249 and the use of insulin during pregnancy (Supplementary Material 1), or having at least 3 claims with ICD-10 codes of O244 or O294.

### Statistical analysis

Continuous variables were represented as the mean± standard deviation, while categorical variables were shown as numbers (percentages). The Student t-test and the chi-square test were employed to compare continuous and categorical variables, respectively. Logistic regression was utilized to calculate the odds ratios (ORs) and 95% confidence intervals (CIs) for incident GDM during pregnancy. The adjusted variables were chosen from baseline characteristics and traditional risk factors for gestational diabetes, which included age, primipara, body mass index (BMI), hypertension, systolic blood pressure and diastolic blood pressure, aspartate aminotransferase, alanine aminotransferase, fasting blood glucose, and total cholesterol. Subgroup analyses were conducted for age and BMI at the onset of pregnancy, as well as for a history of hypertension prior to pregnancy. A p-value of less than 0.05 was deemed statistically significant. All statistical analyses were conducted using SAS version 9.4 (SAS Institute Inc., Cary, NC, USA).

### Ethics statement

The study received approval from the Institutional Review Board of Korea University Guro Hospital (IRB No. 2018GR0403). The need for informed consent was waived, as the NHIS provides anonymized data.

## RESULTS

### Baseline characteristics

The baseline characteristics of the study population are shown in Table 2. The final analysis included a total of 365,880 women. Among these, 3,253 (0.9%) were diagnosed with cardiac arrhythmia, which included premature beats (n= 2,652), paroxysmal tachycardia (n= 478), and atrial fibrillation (n= 159). Women diagnosed with cardiac arrhythmia were, on average, older at the onset of pregnancy compared to those without cardiac arrhythmia (mean age, 33.3± 4.1 vs. 33.0± 3.9 years, p< 0.001). Furthermore, the frequency of cardiac arrhythmia was higher in women aged 35 years and above (33.0 vs. 37.2%, p< 0.001). The prevalence of hypertension was also higher in women with cardiac arrhythmia (1.0 vs. 5.2%, p< 0.001). However, there was no significant difference in BMI (21.4± 3.1 vs. 21.5± 3.2 kg/m^2^, p= 0.386) or in the proportion of first-time mothers (primipara) (70.3 vs. 70.9%, p= 0.435).

### Primary outcome; gestational diabetes

**Figure 1.**
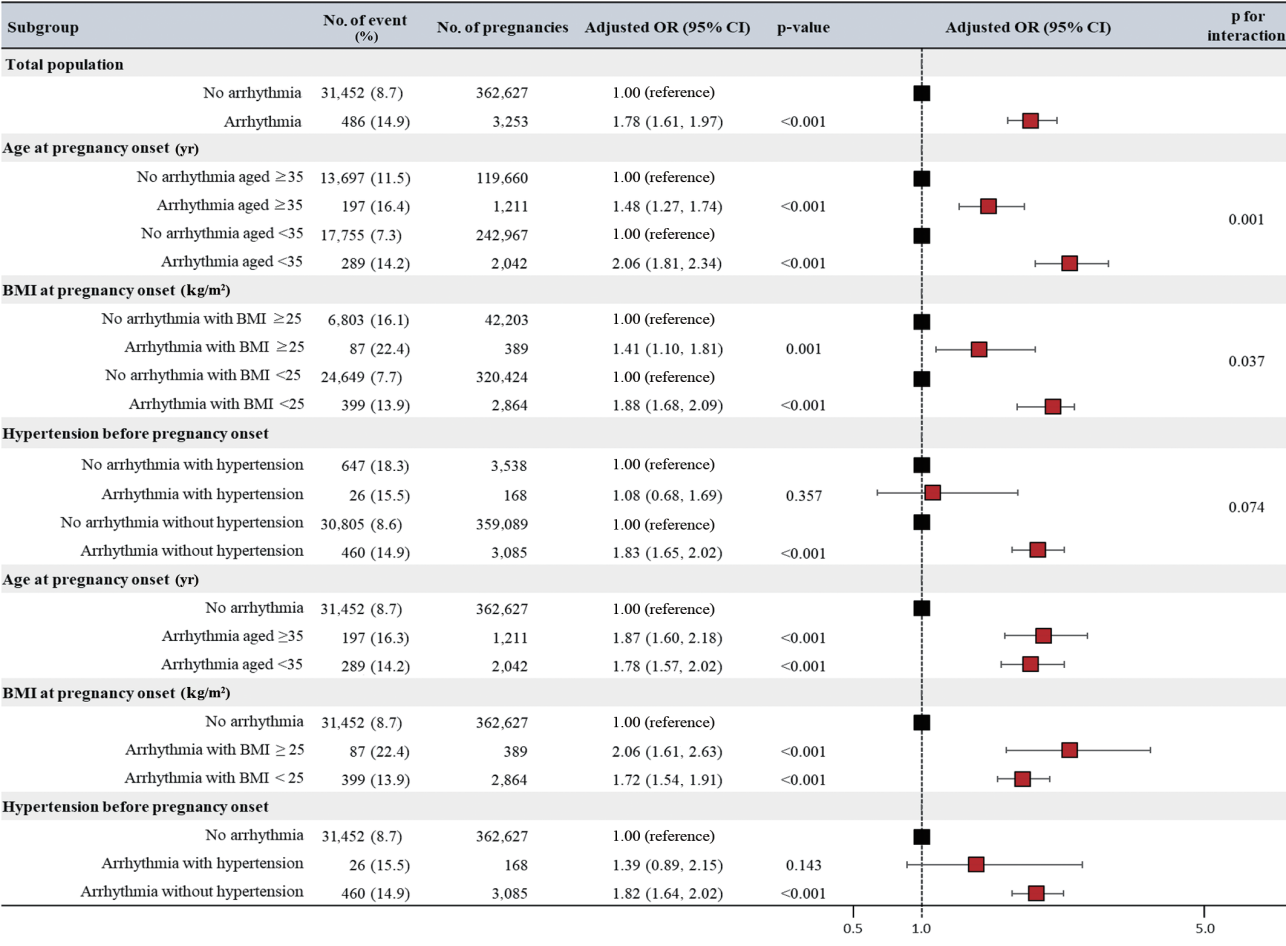
Forest plot of incident gestational diabetes. The dashed vertical line represents the odds ratio (OR) and 95% confidence interval (CI) of incident gestational diabetes in singleton pregnancies without a history of diabetes with cardiac arrhythmia compared to those without cardiac arrhythmia in different subgroups. BMI, body mass index.

During the study period, GDM occurred in 31,938 (8.7%) women. Compared to women without cardiac arrhythmia, those with cardiac arrhythmia were found to have a higher incidence of GDM (8.7 vs. 14.9%, p< 0.001). Even after adjusting for factors such as age, primiparity, BMI, hypertension, systolic blood pressure and diastolic blood pressure, aspartate aminotransferase, alanine aminotransferase, fasting blood glucose, and total cholesterol, the correlation between a history of cardiac arrhythmia and GDM remained statistically significant (adjusted OR, 1.78; 95% CI, 1.61 to 1.97; p< 0.001; Figure 1).

### Subgroup analysis

In the subgroup analysis by age and BMI at the onset of pregnancy, there was a tendency for a higher risk of GDM in subjects with cardiac arrhythmias, mirroring the original analysis (Figure 1). All subjects with cardiac arrhythmia demonstrated a higher risk of developing GDM than those without cardiac arrhythmia. However, the correlation between cardiac arrhythmia and incident GDM was significantly stronger in women under 35 years of age (p for interaction= 0.001) and those with a BMI of less than 25 kg/m^2^ (p for interaction= 0.037). Conversely, no statistically significant interaction was observed in the association between cardiac arrhythmia and incident GDM in relation to the presence or absence of hypertension.

## DISCUSSION

In this study, we found that women with a history of cardiac arrhythmia had an elevated risk of incident GDM during pregnancy. Our results showed that women with cardiac arrhythmia, which includes conditions such as premature beats, paroxysmal tachycardia, and atrial fibrillation/flutter, had a risk of developing GDM that was 1.8 times higher. This correlation was consistently observed across all subjects, irrespective of known risk factors like advanced age, obesity, and hypertension. Moreover, the link between cardiac arrhythmias and the onset of GDM was more pronounced in younger and/or non-obese women.

Premature beats typically do not cause symptoms or hemodynamic disturbances, yet they are linked with an increased risk of cardiovascular morbidity and mortality [9,10]. Traditional risk factors for cardiovascular diseases, such as hypertension [11], obesity [12], sleep apnea [13], and inflammation [14], are associated with ventricular premature beats. Both atrial premature beats and supraventricular tachyarrhythmia are connected to psychological stress [15] or excessive alcohol consumption [16]. Furthermore, a reciprocal relationship exists between atrial fibrillation and diabetes mellitus, although the underlying mechanism for these complications remains unclear [17].

The autonomic nervous system plays a major role in cardiac arrhythmia [18]. Over-activity of the sympathetic system is linked to insulin resistance, which can lead to impaired glucose metabolism and autonomic dysfunction [19]. Duncan et al. [20] also reported that cytokines, such as tumor necrosis factor-alpha and interleukin-1β, can increase susceptibility to ventricular arrhythmia. This suggests that inflammatory processes may contribute to the development of cardiac arrhythmia [20]. Chronic low-grade systemic inflammation is a predictor of both GDM and type 2 diabetes mellitus [21,22]. Other potential common factors include changes in glucose levels, alterations in the sympathetic and/or parasympathetic system, and mitochondrial dysfunction [7]. Therefore, the link between cardiac arrhythmia and GDM could be attributed to the comorbidities and systemic conditions of patients with cardiac arrhythmia, which may heighten their vulnerability to developing GDM.

Recently, it has become increasingly apparent that diabetes mellitus affects the heart’s electrical conduction system, leading to cardiac arrhythmia. This is significant because the primary cardiovascular complication linked with diabetes is coronary artery disease [7]. Consequently, these findings indicate that additional large-scale prospective studies are necessary to verify the relationship between specific cardiac arrhythmias and the risk of GDM.

This retrospective cohort study using a claims database has several limitations. A notable challenge is the absence of detailed clinical data, such as electrocardiogram results, Holter monitoring findings, and physician notes. This limitation primarily relates to the possibility of underdiagnosis or misdiagnosis. Nevertheless, it is essential to highlight that despite the possibility of underdiagnosis, our study has successfully identified a statistically significant association between cardiac arrhythmia and GDM.

In conclusion, cardiac arrhythmia, both before and during pregnancy, appears to be associated with a higher risk of incident GDM. Moreover, the association of cardiac arrhythmia with GDM was statistically significant regardless of well-established risk factors including advanced age, BMI at pregnancy onset, and chronic hypertension.

## Figures and Tables

**Figure f2-epih-45-e2023103:**
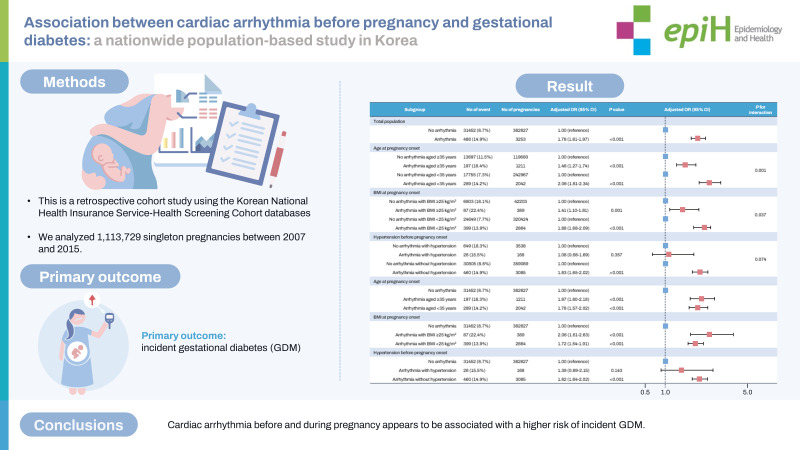


**Table 1.** Categories of arrhythmias and ICD-10 codes

ICD-10 codes	Arrhythmia
Lethal arrhythmia	
I47.2	Ventricular tachycardia
I49.0	Ventricular fibrillation and flutter
I49.01	Ventricular fibrillation
I49.01	Ventricular flutter
Premature beats	
I49.1	Atrial premature depolarization
I49.2	Junctional premature depolarization
I49.3	Ventricular premature depolarization
I49.40	Other and unspecified premature depolarization
I49.49	Other premature depolarization
I49.9	Cardiac arrhythmia, unspecified
Paroxysmal tachycardia	
I47	Paroxysmal tachycardia
I47.0	Re-entry ventricular arrhythmia
I47.1	Supraventricular tachycardia
Atrial flutter/fibrillation	
I48	Atrial fibrillation and flutter
Atrioventricular block	
I44.0	Atrioventricular block, first degree
I44.1	Atrioventricular block, second degree
I44.2	Atrioventricular block, complete

ICD-10, International Classification of Diseases, 10th revision.

**Table 2.** Baseline characteristics of the study population according to the presence or absence of a history of cardiac arrhythmia

Characteristics	No arrhythmia (n=362,627)	Arrhythmia (n=3,253)	p-value
Age at pregnancy onset (yr)	33.0±3.9	33.3±4.1	<0.001
Age ≥35 yr at pregnancy onset	119,660 (33.0)	1,211 (37.2)	<0.001
Systolic blood pressure	110.4±11.0	111.0±11.2	0.001
Diastolic blood pressure	69.2±8.3	69.6±8.6	0.019
Body mass index (kg/m^2^)	21.4±3.1	21.5±3.2	0.386
Body mass index (≥25 kg/m^2^)	42,203 (11.6)	389 (12.0)	0.571
Current smoker	28,012 (7.7)	234 (7.2)	0.258
Primipara	254,782 (70.3)	2,306 (70.9)	0.435
Hypertension	3,538 (1.0)	168 (5.2)	<0.001
Aspartate aminotransferase (IU/L)	19.3±13.1	19.1±7.0	0.400
Alanine aminotransferase (IU/L)	15.3±20.4	15.3±11.6	0.911
Fasting blood glucose (mg/dL)	88.7±9.0	89.2±9.2	0.003
Total cholesterol (mg/dL)	182.2±32.2	182.5±32.0	0.546

Values are presented as mean±standard deviation or number (%).

## SUPPLEMENTARY MATERIALS

Supplementary Material 1. Domestic codes for insulin used in the Korean National Health Insurance Service database
