# Supplementary material for: Association between cardiac arrhythmia before pregnancy and gestational diabetes: a nationwide population-based study in Korea
Source: Epidemiol Health. 2023 Dec 4;45:e2023103. doi: 10.4178/epih.e2023103 (PMC10876443; doi:10.4178/epih.e2023103)
Supplement: Supplementary Material 1. — Domestic codes for insulin used in the Korean National Health Insurance Service database [file epih-45-e2023103-Supplementary-1.pdf]

Supplemental Table 1. Domestic codes for insulin used in the Korean National Health Insurance Service database

Medication	Codes
Insulin	118301BIJ, 170101BIJ, 170102BIJ, 170103BIJ, 170130BIJ, 170131BIJ, 170201BIJ, 170302BIJ, 170401BIJ, 170402BIJ, 170403BIJ, 170430BIJ, 170431BIJ, 170501BIJ, 170502BIJ, 170602BIJ, 175201BIJ, 175202BIJ, 175301BIJ, 175302BIJ, 175303BIJ, 175304BIJ,175330BIJ, 175331BIJ, 175332BIJ, 175333BIJ, 215602BIJ, 215603BIJ, 215701BIJ, 441301BIJ, 441302BIJ, 441303BIJ, 441304BIJ, 441305BIJ, 441330BIJ, 441331BIJ, 441332BIJ, 441333BIJ, 441334BIJ, 461801BIJ, 461802BIJ, 461804BIJ, 461830BIJ, 461831BIJ, 461832BIJ, 484901BIJ, 484902BIJ, 484930BIJ, 484931BIJ, 488701BIJ, 488730BIJ, 507401BIJ, 626700BIJ, 626801BIJ, 626802BIJ, 626830BIJ, 626831BIJ, 666700BIJ, 667000BIJ
